# Metabolic Dysfunction-Associated Steatotic Liver Disease in People with Type 1 Diabetes

**DOI:** 10.3390/jcm14155502

**Published:** 2025-08-05

**Authors:** Brynlee Vermillion, Yuanjie Mao

**Affiliations:** 1 Erie Campus, Lake Erie College of Osteopathic Medicine, Erie, PA 16508, USA; bvermillio60475@med.lecom.edu; 2 Diabetes Institute, Ohio University Heritage College of Osteopathic Medicine, Athens, OH 45701, USA; 3 Endocrinology & Diabetes Clinic, OhioHealth Castrop Health Center, Athens, OH 45701, USA

**Keywords:** metabolic dysfunction-associated steatotic liver disease, type 1 diabetes, insulin resistance, complications

## Abstract

Metabolic dysfunction-associated steatotic liver disease (MASLD) is increasingly recognized as a significant comorbidity in individuals with type 1 diabetes (T1D), despite its historical association with type 2 diabetes. This review focuses on summarizing current findings regarding the role of insulin resistance in the development of MASLD in T1D, as well as examining the relationship between MASLD and diabetes-related complications. We will also briefly discuss the prevalence, diagnostic challenges, associated complications, and potential mechanisms underlying MASLD in T1D. Although insulin resistance is well established in MASLD among those with type 2 diabetes, its role in T1D requires further clarification. Emerging markers, such as the estimated glucose disposal rate, offer early insight into this relationship. MASLD in T1D is linked to both microvascular and macrovascular complications, including nephropathy, retinopathy, neuropathy, and cardiovascular disease. Variability in prevalence estimates reflects inconsistencies among imaging modalities, emphasizing the need for standardized, non-invasive diagnostic approaches. Recognizing and addressing MASLD and its links to insulin resistance and diabetes complications in T1D is vital for mitigating long-term complications and enhancing clinical outcomes.

## 1. Introduction

Metabolic dysfunction-associated steatotic liver disease (MASLD), formerly known as non-alcoholic fatty liver disease (NAFLD), is characterized by the accumulation of fat in the liver in the absence of significant alcohol consumption. It has become the most prevalent chronic liver condition worldwide, affecting an estimated 25% of the global adult population. With its rising prevalence, MASLD is now recognized as a major contributor to liver-related morbidity and mortality [1]. The disease spectrum ranges from simple hepatic steatosis, which often follows a benign course, to non-alcoholic steatohepatitis (NASH), a more severe form involving hepatic inflammation and fibrosis. In advanced stages, MASLD may progress to cirrhosis, liver failure, and hepatocellular carcinoma [2].

Beyond liver-specific complications, MASLD is increasingly linked to systemic conditions such as metabolic syndrome, chronic kidney disease (CKD), and cardiovascular disease (CVD) [3]. The pathogenesis of MASLD is complex and multifactorial, with insulin resistance playing a central role. Insulin resistance promotes an increase in lipolysis, free fatty acid influx to the liver, and de novo lipogenesis—all of which contribute to hepatic fat accumulation. It also exacerbates oxidative stress and inflammatory signaling, accelerating progression from simple steatosis to NASH and fibrosis. Additional contributors include oxidative stress and dysregulation of lipid metabolism [4]. The gold standard for diagnosing MASLD remains liver biopsy, which provides histological confirmation of the disease. A histological diagnosis requires the presence of hepatic steatosis in at least 5% of hepatocytes without evidence of hepatocellular injury [1]. However, due to the invasive nature of biopsy, many patients prefer non-invasive diagnostic methods. These include imaging techniques such as ultrasound, computed tomography (CT), and magnetic resonance imaging (MRI), which can assess liver fat content and aid in diagnosis.

Type 1 diabetes (T1D) is an autoimmune disorder characterized by immune-mediated destruction of pancreatic beta cells, leading to insulin deficiency. It typically manifests in childhood or adolescence and is associated with hyperglycemia, ketosis, and the presence of ketone bodies. Individuals with a family history of T1D are at increased risk, although the general population risk is approximately 0.4%. The disease progresses through three stages. In stage one, individuals are asymptomatic with normoglycemia, despite the beginning of beta-cell destruction. Stage two involves mild hyperglycemia without overt symptoms. Most diagnoses occur at stage three, marked by significant hyperglycemia and clinical symptoms such as polydipsia, polyuria, dehydration, and weight loss [5].

Type 2 diabetes (T2D), in contrast, arises from a combination of insulin resistance and beta-cell dysfunction. It is influenced by both genetic and environmental factors, with obesity—particularly visceral fat accumulation—being a major risk factor. Visceral adiposity, especially in the abdominal region around organs like the liver and intestines, correlates with greater insulin resistance [6]. Clinically, patients with T1D tend to be younger and leaner than those with T2D. C-peptide levels are often used to distinguish the two: low levels in T1D reflect impaired insulin production, whereas levels may remain normal or elevated in T2D [7].

While MASLD is traditionally associated with T2D due to its strong links with obesity and metabolic syndrome, emerging evidence suggests its prevalence among individuals with T1D is growing. This is concerning, as MASLD in T1D has been associated with a higher risk of liver fibrosis, microvascular complications, and increased cardiovascular risk, despite the absence of obesity in many patients. These findings challenge conventional assumptions and underscore the importance of investigating the pathophysiology and clinical consequences of MASLD in T1D.

Accordingly, this review aims to summarize current knowledge regarding the role of insulin resistance in the development of MASLD in T1D and to examine the associations between MASLD and diabetes-related complications in this population. To achieve this, a comprehensive literature search was conducted in PubMed using the following keywords: type 1 diabetes, insulin resistance, and NAFLD/MASLD. The search returned approximately 53 articles. However, after excluding review articles and animal studies, and screening titles and abstracts for relevance to human subjects with type 1 diabetes, 25 original research articles were included for full-text evaluation.

A second focused search was also conducted to examine the relationship between MASLD and diabetes-related complications in individuals with T1D. Using the keywords type 1 diabetes, NAFLD/MASLD, and complications (including microvascular and macrovascular outcomes), approximately 149 articles were initially identified. After applying the same exclusion criteria and screening process, 22 original articles were selected for full-text review.

## 2. Prevalence and Risk Factors

Although T1D and T2D differ in etiology and clinical presentation, they share overlapping features, particularly as the prevalence of obesity increases among individuals with T1D—a trait historically associated with T2D [8]. Given the rising burden and mortality associated with MASLD, it is critical to assess its prevalence among individuals with T1D as these shared features become more apparent.

Estimates of MASLD prevalence in T1D vary widely depending on the diagnostic modality employed. In a 2010 study, Targher et al. utilized liver ultrasound to assess MASLD in individuals with pre-existing T1D and reported a prevalence of 44.4%, identifying a significant association between the two conditions [9]. Similarly, another study examining the link between CKD and T1D found MASLD in 131 of 234 participants (56.0%) [10]. In contrast, other studies report a lower prevalence range of 10–25% in individuals with T1D. This is notably lower than the 55–70% prevalence reported among those with T2D [11]. Furthermore, a 2014 study found that 50% of individuals with T1D and MASLD demonstrated hepatic steatosis, despite most exhibiting liver enzyme levels within the normal range [10], suggesting that current liver function tests lack the sensitivity to detect MASLD reliably. Variability in reported prevalence may also be due to underdiagnosis, stemming from the historical focus on MASLD in T2D populations.

To investigate these discrepancies, De Vries et al. conducted a meta-analysis evaluating 20 studies comprising 3901 individuals with T1D, published between 2009 and 2019. The study population ranged in age from 12.9 to 48.9 years. The overall prevalence of MASLD was reported as 19.3% of all participants and 22.0% among adults [8]. Importantly, the study highlighted significant variation based on diagnostic methodology, including ultrasound, MRI, liver biopsy, and multimodal approaches, with ultrasound appearing to yield higher detection rates compared to MRI.

Although ultrasound is widely used for assessing MASLD, particularly in epidemiological studies, it presents important limitations, especially in lean, pediatric, and young adult T1D populations. Its accuracy is highly operator-dependent and has reduced sensitivity and specificity (approximately 73% sensitivity and 70% specificity) for detecting moderate-to-mild steatosis compared with histology, and performance declines further when hepatic fat content is low [12]. MRI/MRS provides a more accurate and reproducible quantification of liver fat. However, these modalities are costly, time-consuming, and may be less practical for routine clinical use in younger patients or environments with limited resources [13]. While liver biopsy remains definitive, it is impractical for widespread application in the pediatric population due to its invasive nature. Serum-based fibrosis scores such as FIB-4 and NAFLD Fibrosis Score (NFS) show promise for identifying advanced fibrosis in adults, and FIB-4 thresholds may be optimized in diabetes to improve sensitivity [14]. However, these scores have yet to be validated in children or adolescents with T1D, wherein age-related differences in liver enzymes and platelet counts may limit their accuracy [15]. These considerations emphasize the need for diagnostic tools that balance sensitivity, feasibility, and clinical applicability in young people with T1D and MASLD.

In another study, Mertens et al. aimed to clarify prevalence and risk factors by employing magnetic resonance spectroscopy (MRS), a more precise imaging modality. Their findings indicated a MASLD prevalence of 15.9%, with 21 of the enrolled patients testing positive [16]. Male sex and obesity were identified as more common among MASLD cases [16], supporting the theory that MASLD in T1D is influenced by metabolic syndrome components. Among the most consistent risk factors for MASLD in T1D is poor glycemic control, often reflected by elevated glycated hemoglobin (HbA1c) levels [17]. Chronic hyperglycemia may promote hepatic fat accumulation through enhanced de novo lipogenesis, oxidative stress, and mitochondrial dysfunction [9]. Additional metabolic syndrome features—such as central obesity, hypertension, hypertriglyceridemia, and low high-density lipoprotein (HDL) cholesterol levels—are also frequently observed in T1D patients with MASLD [18]. These risk factors are believed to be driven by shifts in dietary habits and reduced physical activity levels, contributing to a rising prevalence of obesity in younger populations [19].

## 3. MASLD and Insulin Resistance in T1D

Insulin resistance may play a central role in the development of MASLD in individuals with T1D. Although T1D is classically defined by absolute insulin deficiency due to autoimmune β-cell destruction, many patients develop insulin resistance over the course of their treatment [11]. Notably, approximately 19.3% of individuals with T1D and 22% of adults with T1D who also exhibit insulin resistance have been reported to develop MASLD [11]. This may be partially attributed to the increasing prevalence of obesity in the T1D population [20]. Furthermore, chronic exposure to exogenous insulin has been implicated in promoting hepatic lipogenesis and increasing intrahepatic fat accumulation, thereby elevating the risk of MASLD [1]. However, despite these observations, research specifically linking T1D, insulin resistance, and MASLD remains limited.

To address this knowledge gap, a 2022 study investigated the association between insulin resistance and MASLD in a non-preselected cohort of patients with T1D. The researchers utilized transient elastography (TE) to assess hepatic steatosis and fibrosis and estimated glucose disposal rate (eGDR) as a surrogate measure of insulin resistance. Among 150 participants with T1D, 30 were found to have hepatic steatosis, while 5 (3.3%) showed evidence of liver fibrosis [20]. The authors concluded that most MASLD cases in this group were due to hepatic fat accumulation without concurrent fibrosis. Importantly, those with greater insulin resistance, as indicated by lower eGDR values, were more likely to have MASLD, highlighting a potential link between insulin resistance and hepatic fat deposition in T1D, although other pathophysiologic mechanisms are also likely to contribute [20]. To provide a broader view of this relationship, Table 1 summarizes key studies investigating the association between insulin resistance and MASLD in individuals with T1D, including study design, diagnostic methods, markers used for insulin resistance, and main findings (Table 1).

Other studies employing eGDR to evaluate the relationship between insulin resistance and MASLD have yielded mixed results. For instance, one study reported a MASLD prevalence of 30% in individuals with T1D but did not find a significant correlation between insulin resistance and hepatic fat content when using MRI as the diagnostic tool [22]. In contrast, Perseghin et al. offered a compelling alternative explanation [23]. Their study focused on individuals with poorly controlled T1D and found that, despite evidence of systemic and hepatic insulin resistance, these patients had *lower* intrahepatic fat content compared to healthy controls. This unexpected result suggested the involvement of additional mechanisms modulating liver fat storage in T1D.

While insulin resistance remains a potential contributor to MASLD in T1D, emerging evidence suggests that additional, T1D-specific mechanisms may also play a significant role. For example, in individuals with T1D, exogenous insulin is delivered subcutaneously, which bypasses the portal circulation and results in lower hepatic insulin exposure compared to physiologic insulin secretion [24]. This altered distribution could impair the liver’s ability to suppress gluconeogenesis and effectively regulate lipid metabolism, contributing to steatosis even in the absence of obesity. Relative hyperglucagonemia—commonly seen in T1D—can increase glucose production in the liver and influence lipid metabolism [23]. Autoimmune-mediated inflammation may further impact hepatocellular function [25], although its precise role in hepatic fat accumulation is not well defined. Several studies have reported hepatic steatosis in lean individuals with T1D [26], suggesting that ectopic fat accumulation in this population may arise through pathways distinct from those observed in T2D. These findings highlight the need to consider alternative models for MASLD development in T1D that extend beyond traditional risk factors.

Perseghin et al. further explored metabolic factors that may explain this paradox. Their study revealed that individuals with T1D had an elevated glucagon-to-insulin ratio and increased levels of non-esterified fatty acids (NEFAs), which promote lipolysis and fatty acid mobilization [23]. Metabolic assessments showed no significant difference in total energy expenditure between T1D patients and controls. However, fuel utilization differed: patients with T1D relied more heavily on lipid oxidation, particularly during fasting or in response to exogenous insulin administration. The elevated glucagon-to-insulin ratio appeared to promote hepatic fatty acid oxidation rather than fat storage, accounting for the observed reduction in intrahepatic lipid accumulation [21]. These observations underscore the complex metabolic adaptations in T1D that influence hepatic lipid processing, independent of traditional obesity-driven insulin resistance.

Importantly, the metabolic dysregulation seen in poorly controlled T1D extends beyond the liver. In skeletal muscle, the perceived “fed” state—due to elevated glucose, amino acids, and NEFAs—leads to the accumulation of malonyl-CoA. This inhibits mitochondrial fatty acid transport and results in lipid deposition within muscle cells as intramyocellular lipids (IMCLs) [27]. The accumulation of IMCLs interferes with insulin signaling, contributing to insulin resistance in muscle tissue. These findings suggest that insulin resistance in T1D may serve as a systemic adaptation to metabolic imbalances rather than being a consequence of hepatic steatosis alone [21].

While the liver in poorly controlled T1D may avoid steatosis due to enhanced lipid oxidation, insulin resistance can still develop in other tissues. The authors propose that individuals with well-controlled T1D may maintain normal hepatic glycogen stores and preserve a physiological glucagon-to-insulin ratio, which could protect against both insulin resistance and MASLD development [21]. Together, these findings suggest that MASLD in T1D cannot be fully explained by insulin resistance alone and is likely to result from a combination of unique factors, including insulin delivery patterns, glucagon dysregulation, autoimmune effects, and ectopic fat metabolism. The convergence of these factors warrants further investigation.

## 4. MASLD and Diabetes Complications in T1D

In individuals with T1D, MASLD has been increasingly associated with the development of both microvascular and macrovascular complications. A summary of studies examining the association between MASLD and diabetes-related complications in individuals with T1D is presented in Table 2. This includes the types of complications assessed, diagnostic modalities used, and major findings. Among them, CKD has been most consistently linked to the presence of MASLD. A longitudinal study assessing T1D patients over a five-year period demonstrated a significantly increased risk of CKD among those with MASLD, independent of other common risk factors such as age, glycemic control, blood pressure, and baseline renal function [10]. The Kaplan–Meier analysis from the longitudinal study revealed a *p*-value of <0.001, suggesting a strong statistical association between MASLD and subsequent development of CKD. These findings were supported by a 2021 study by Mertens et al., which identified a similar increased risk of CKD in patients with both MASLD and diabetes [28]. The authors attributed this risk to the synergistic effects of hyperglycemia and hypertension, which exacerbate glomerular damage and contribute to progressive nephropathy [28].

Beyond CKD, MASLD has also been associated with other microvascular complications, such as diabetic retinopathy and neuropathy. A cross-sectional study utilizing ultrasound for MASLD diagnosis found a markedly higher prevalence of diabetic retinopathy in individuals with T1D and MASLD compared to those without hepatic steatosis—53% versus 20%, respectively [29]. Even after adjusting for confounders such as age, sex, duration of diabetes, HbA1c, and features of metabolic syndrome, the presence of MASLD remained independently associated with more than a threefold increased risk of retinopathy [30].

Although diabetic polyneuropathy is a common complication of T1D, evidence connecting MASLD to nerve damage is relatively sparse. Poor glycemic control remains a major risk factor for neuropathy, but other elements such as dyslipidemia, hypertension, and smoking may also contribute. One study evaluated 286 individuals with T1D and found that 51% of those with MASLD had evidence of distal symmetric polyneuropathy (DSPN), compared to just 17% of those without MASLD. After adjusting for relevant metabolic and clinical factors, MASLD remained associated with more than twice the risk of DSPN [31]. These findings suggest that MASLD may be an underrecognized contributor to diabetic neuropathy in T1D.

**Table 2:** Summary of Studies Investigating Complications Associated with MASLD in Individuals with Type 1 Diabetes.

Author (Year)	Design	Sample Size (n)	MASLD Diagnosis	Complications	Inclusion/Exclusion	Key Findings
Mantovani et al., 2024 [31]	Retrospective, multi-center, cross-sectional	1409	Non-invasive biomarkers; hepatic steatosis index (HSI > 36) and FIB-4 index (≥1.3 for significant fibrosis)	Chronic Kidney Disease (CKD); diabetic retinopathy	Inclusion: Adults with T1D; Exclusion: Not specified	MASLD with fibrosis was associated with higher risk of CKD (aOR 1.76; 95% CI 1.05–2.96); MASLD without fibrosis was associated with higher risk of diabetic retinopathy (aOR 1.49; 95% CI 1.13–1.46); The findings suggest that MASLD is independently associated with microvascular complications in T1D.
Fuhri Snethlage et al., 2024 [32]	Cross-sectional, observational	453	Vibrationcontrolled transient elastography (VCTE); MASLD defined by CAP score ≥ 280 dB/m and fibrosis by LSM ≥ 8.0 kPa	Cardiovascular disease (CVD); microvascular complications; high blood pressure	Inclusion: Adults with T1D; Exclusion: Not specified	Prevalence of steatosis: 9.5%, fibrosis: 3.5%. Fibrosis was associated with older age, longer diabetes duration, higher BMI, and systolic BP. Machine learning identified duration of diabetes, age, and systolic BP as top predictors.
Maffeis et al., 2024 [33]	Cross-sectional, observational	244	Hepatic steatosis defined by ultrasound, plus ≥ 1 cardiometabolic risk factor (per MASLD definition)	Cardiovascular disease (CVD)	Inclusion: Children and adolescents with T1D; Exclusion: Not specified	MASLD prevalence was 27.5%. Higher HbA1c (from onset), time above range (TAR), and LDL cholesterol were independently associated with MASLD. The findings suggest that poor glycemic control and dyslipidemia contribute to MASLD risk in pediatric T1D patients.
Targher et al., 2010 [9]	Cross-sectional	250	History + liver ultrasound	Cardiovascular disease (coronary, cerebrovascular, and peripheral)	Inclusion: T1D patients with liver ultrasound data, attending diabetes clinic regularly; Exclusion: Not specified	MASLD prevalence was 44.4%. MASLD was independently associated with higher prevalence of coronary (10.8% vs. 1.1%), cerebrovascular (37.3% vs. 5.5%), and peripheral (24.5% vs. 2.5%) vascular disease. Adjusted OR for CVD = 7.36.
Targher et al., 2010 [34]	Cross-sectional, biopsy-based, case-control	160 (80 NASH + 80 controls)	Liver biopsy (NASH); the control group had no steatosis	Chronic Kidney Disease (CKD); low eGFR; albuminuria	Inclusion: Overweight adults matched by age, sex, BMI; 80 with biopsy-proven NASH, 80 controls without steatosis; Exclusion: Not specified	NASH patients had lower eGFR (75.3 vs. 87.5), more albuminuria (14% vs. 2.5%), and higher CKD prevalence (25% vs. 3.7%). Differences remained significant after adjusting for insulin resistance, metabolic syndrome, and other factors. Severity of NASH (fibrosis) correlated with worse kidney function.
Mantovani et al., 2017 [35]	Retrospective, cross-sectional	286	Ultrasonography	Distal symmetric polyneuropathy	Inclusion: White adults with T1D attending foot screening; Exclusion: Excess alcohol intake and other known liver diseases	MASLD was present in 52.4% of participants. MASLD was associated with significantly higher prevalence of polyneuropathy (51.0% vs. 17.1%). Adjusted OR for polyneuropathy = 2.23, independent of glycemic control, CKD, and metabolic risk factors.
Serra-Planas et al., 2017 [36]	Cross-sectional	100	Abdominal ultrasonography	Subclinical CVD (↑ CIMT, CACS > 0, carotid plaques)	Inclusion: Adults with T1D undergoing abdominal + carotid ultrasound and cardiac CT; Exclusion: Not specified	MASLD prevalence was 12%. T1D subjects with MASLD had higher CIMT (0.65 ± 0.17 vs. 0.55 ± 0.14 mm; *p* = 0.029) but no significant differences in CACS or plaque presence. Elevated liver enzymes (>20 U/L) were also linked to worse imaging markers.
Zhang et al., 2023 [37]	Cross-sectional	12,990	Ultrasound	Carotid plaque	Inclusion: Chinese aged adults; Exclusion: Excess alcohol intake (men > 210 g/week, women > 140 g/week); censored for elevated ALT (≥75 IU/L), CVD, and obesity in sensitivity analyses.	MASLD was associated with higher carotid plaque prevalence (22.4% vs. 16.3%). Adjusted OR = 1.89 (95% CI: 1.59–2.24). Association remained significant after adjusting for multiple metabolic and clinical variables and after sensitivity analyses.

Note: MASLD = metabolic dysfunction-associated steatotic liver disease; T1D = type 1 diabetes.

In addition to microvascular disease, MASLD in T1D has also been implicated in the development of macrovascular complications, most notably CVD. While the association between MASLD and early atherosclerosis has been well-documented in T2D, fewer studies have explored this relationship in T1D. However, one study of 722 individuals with T1D found that patients with MASLD had significantly thicker carotid intima-media layers and a higher prevalence of carotid plaques (28.9%) compared to those without MASLD (16.9%). Age, MASLD, and markers of systemic inflammation were each independently associated with increased arterial thickness [9].

Despite this growing body of evidence linking MASLD to cardiovascular complications, its impact on CVD-related mortality remains uncertain [30]. Some studies report no increased risk of CVD death in individuals with MASLD, while others find a significantly elevated mortality risk. Notably, the latter studies typically focus on patients with more advanced liver disease, particularly those with hepatic fibrosis. This suggests that the severity of MASLD—rather than its mere presence—may be a critical determinant of cardiovascular outcomes. Indeed, liver fibrosis has emerged as the most robust predictor of fatal cardiovascular events in these populations [30].

Discrepancies among studies may be due to differences in diagnostic criteria, the imaging modalities used to detect MASLD, and variation in patient characteristics, including comorbid conditions and prior diabetes management. As a result, further prospective studies using standardized MASLD definitions and diagnostic methods are needed to clarify the extent of its role in diabetes-related complications.

## 5. Conclusions

MASLD is an increasingly recognized comorbidity in individuals with T1D, with growing evidence suggesting that its prevalence may be significantly underestimated. This review summarizes the current literature linking MASLD to T1D, focusing specifically on the role of insulin resistance in its development and the associations between MASLD and diabetes-related complications.

A key diagnostic challenge lies in the fact that many individuals with MASLD exhibit normal liver enzyme levels, underscoring the need for more sensitive and non-invasive screening methods. The wide variability in reported prevalence is largely due to inconsistencies in diagnostic modalities—such as ultrasound, MRI, and MRS—as well as differences in study design, diagnostic thresholds, and patient populations.

While the role of insulin resistance in MASLD is well characterized in T2D, its significance in T1D is less clear. Emerging studies utilizing surrogate markers such as eGDR suggest a potential link between insulin resistance and MASLD in T1D. However, these findings remain preliminary and call for validation using standardized, reliable diagnostic approaches.

This review also highlights the association between MASLD and both microvascular complications—including chronic kidney disease, retinopathy, and neuropathy—and macrovascular complications, such as cardiovascular disease. These associations suggest that MASLD may serve as a marker of broader metabolic dysfunction and could contribute significantly to long-term morbidity in individuals with T1D.

Improved understanding of insulin resistance and the spectrum of complications linked to MASLD in T1D is critical to developing targeted screening and management strategies, with the ultimate goal of reducing disease burden and improving outcomes in this population.

## 6. Future Directions

To better characterize the role of MASLD in the context of T1D, there is a critical need for focused, hypothesis-driven research. Large-scale, prospective cohort studies—particularly those stratified by insulin delivery method (e.g., multiple daily injections vs. insulin pump therapy)—may help determine whether differences in insulin distribution affect MASLD development. Consistent use of standardized diagnostic criteria will be essential to accurately define the prevalence and history of MASLD in this population.

The use of validated, non-invasive tools—such as elastography, MRI-based techniques, and serum biomarkers—may improve detection and allow for more precise assessment of disease severity, particularly in lean or pediatric patients, wherein traditional ultrasound has limitations. Emerging biomarkers, including FIB-4 index and NAFLD Fibrosis Score, show promise for early risk stratification, but their performance in T1D-specific populations remains to be established.

Additionally, more work is needed to define the underlying mechanisms contributing to MASLD in T1D. While insulin resistance appears to play a role, additional factors—such as altered hepatic insulin signaling, glucagon dominance, adipose tissue dysfunction, and autoimmune inflammation—may contribute in this setting. Studies investigating inflammatory and metabolic markers specific to T1D could provide insight into whether MASLD in this population follows a distinct pathophysiological trajectory compared to T2D.

Finally, interventional studies are needed to explore the potential treatment strategies for MASLD in individuals with T1D. Lifestyle modification—including nutrition counseling, physical activity, and weight optimization—remains the foundational approach. However, clinical trials evaluating the effects of pharmacologic agents—such as GLP-1 receptor agonists and SGLT2 inhibitors—on liver fat and fibrosis in T1D would be valuable. GLP-1 receptor agonists and SGLT2 inhibitors, although not currently approved for MASLD treatment in T1D, have shown benefits in reducing hepatic steatosis and improving metabolic parameters in T2D and may offer future therapeutic options [33]. Investigating whether early identification and management of MASLD can help reduce the risk of vascular and renal complications could be key in improving long-term outcomes for this patient population.

## Figures and Tables

**Table 1:** Summary of Studies Examining the Association Between Insulin Resistance and MASLD in Individuals with Type 1 Diabetes.

Author (Year)	Design	Sample Size (n)	MASLD Diagnosis	Markers for Insulin Resistance	Inclusion/Exclusion	Key Findings
Grezelka-Woźniak et al., 2023 [21]	Cross-sectional, observational	151	Transient elastography (TE); MASLD defined as controlled attenuation parameter (CAP) ≥238 dB/m	Estimated Glucose Disposal Rate (eGDR); Visceral Adiposity Index (VAI); Triglyceride-to-HDL- Cholesterol Ratio (TG/HDL-C)	Inclusion: Adults with T1D; Exclusion: Not specified	MASLD was present in 43% of patients; indirect insulin resistance markers were independently associated with MASLD
de Vries et al., 2024 [22]	Cross-sectional, observational	254 (150 with TE)	Transient elastography (TE); MASLD defined via imaging-based assessment	Estimated Glucose Disposal Rate (eGDR)	Inclusion: Adults with T1D from secondary/tertiary care centers. Exclusion: Not specified	No dose-dependent association found between physical activity and insulin resistance/MASLD. Sports participation was significantly associated with higher insulin sensitivity (eGDR) and lower odds of MASLD (OR 0.21; 95% CI 0.08–0.56).
de Vries et al., 2022 [20]	Prospective, observational	150	Transient elastography (TE); MASLD defined as hepatic steatosis (HS) with or without fibrosis/cirrhosis	Estimated Glucose Disposal Rate (eGDR); metabolic syndrome	Inclusion: Adults with T1D; Exclusion: Known secondary causes of liver disease	MASLD was present in 20% of patients; 3.3% had fibrosis; lower eGDR (OR 0.62; 95% CI 0.49–0.77) and presence of metabolic syndrome (OR 7.62; 95% CI 2.95–19.77) were significantly associated with MASLD, suggesting that insulin resistance is a key risk factor

Note: MASLD = metabolic dysfunction-associated steatotic liver disease; T1D = type 1 diabetes.

## Data Availability

No new data were created or analyzed in this study. Data sharing is not applicable to this article.
